# Multi-level validation of the German physical activity self-efficacy scale in a sample of female sixth-graders

**DOI:** 10.1186/s12889-020-09096-4

**Published:** 2020-06-22

**Authors:** Joachim Bachner, David J. Sturm, Stephan Haug, Yolanda Demetriou

**Affiliations:** 1 Department of Sport and Health Sciences, Technical University of Munich, Georg-Brauchle-Ring 62, 80992 Munich, Germany; 2 Department of Mathematics, Technical University of Munich, Boltzmannstraße 3, 85748 Garching, Germany

**Keywords:** Multi-level, Lavaan, Confirmatory factor analysis, Internal consistency, Criterion validity, Adolescents

## Abstract

**Background:**

The majority of children and adolescents are insufficiently physically active. Self-efficacy is considered one of the most important determinants of physical activity (PA). The purpose of this study was to validate the German version of the physical activity self-efficacy scale by means of a multi-level approach. Factorial validity, internal consistency and criterion validity were examined for the individual and the class level.

**Methods:**

The final sample comprised 454 female sixth-graders of 33 classes. To examine the factorial validity of the translated 8-item scale, a multi-level confirmatory factor analysis was conducted with the lavaan package in R. Internal consistency was estimated with the alpha function of the psych package. Criterion validity was examined by correlating self-efficacy with moderate-to-vigorous physical activity (MVPA) assessed with accelerometers.

**Results:**

In contrast to previous validation studies, a unidimensional structure of the scale was not supported. Instead, two highly correlated (r_individual_ = .87; r_class_ = .69) but distinct latent factors, representing PA self-efficacy and social support from family and friends, were differentiated on both the individual and class level. The best overall fit exhibited a multi-level 1 × 1-model, including only the six items measuring PA self-efficacy (χ2 = 32.10, CFI = .986, TLI = .976, RMSEA = .059, SRMR = .035). Internal consistencies for the complete 8-item scale and the 6-item scale were good on the individual level and excellent on the class level. For the two items measuring social support, Cronbach’s alpha was low on the individual and excellent on the class level. Weak relations between self-efficacy and MVPA were found for the individual level, strong associations were found for the class level.

**Conclusions:**

The validation speaks for the use of the abridged 6-item scale, which allows for a unidimensional assessment of PA self-efficacy. Generally, the results support the relevance of a multi-level approach, which not only differentiates between self-efficacy on the individual level and on the class level but also between the respective implications regarding reliability and criterion validity on both levels. Thereby, this study offers a rigorously validated scale and further illustrates possible consequences of the usual neglect of group-level variance in scale validation.

## Background

Regular physical activity (PA) contributes to the prevention of chronic diseases, such as diabetes mellitus, cancer or cardiovascular diseases, and lowers the risk of premature death [1–4]. The World Health Organization (WHO) recommends children and youths aged 5 to 17 years to accumulate at least 60 min of moderate-to-vigorous physical activity (MVPA) per day, with MVPA comprising any type of activity that requires at least as much energy as is spent during ordinary walking [5]. There are two reasons why it is important for children and adolescents fulfil the PA recommendation. One would be the positive short- and middle-term effects on their health and well-being [1, 6–8]. Another reason would be a tracking effect that describes the role of adolescents’ PA as a significant predictor of PA in adulthood: The more active a person is in adolescence, the higher the probability of an active lifestyle in adulthood [7, 9].

According to a questionnaire-based study, only 26% of children and adolescents in Germany aged between 3 and 17 years reach the daily 60 min of MVPA. Furthermore, less girls than boys (22.4% vs. 29%) fulfil this recommendation [10]. In addition, PA levels in this population decline with increasing age [10, 11]. A systematic review of Van Hecke et al. [12] supports the effects of gender and age on PA. Although not even the most popular device-based approaches like accelerometry offer perfectly reliable PA data [12–14], a vast majority of studies indicates that PA in adolescence does not comply with the respective recommendation [15, 16]. Moreover, the WHO recommendation is merely regarded as a minimum value. Higher MVPA levels are associated with additional health benefits [17]. Therefore, in any case it is worthwhile to promote PA from an early age.

At this point, the question arises which determinants should be focused on to increase youth’s PA. Ecological models suggest that PA is affected by several interacting levels of influence ranging from policy variables, such as investments in public recreation facilities, to intrapersonal variables, including psychological constructs [18]. Among these psychological constructs, self-efficacy concerning PA is of great importance. In a review of reviews by Bauman and colleagues [19], self-efficacy was the only psychological factor consistently identified as a positive correlate and determinant of PA in children and adolescents. This finding was confirmed by an umbrella systematic review specifically focussing on psychological constructs [20]. Yet another systematic review [21] focused on the PA-related age effect and indicated that self-efficacy was one of very few constructs able to reduce the decline in PA between the age of ten and 18 years. Furthermore, two systematic reviews [22, 23] analysing intervention studies identified PA self-efficacy as the most promising mediator to increase PA.

Due to its high relevance, self-efficacy has been extensively examined in the field of PA. Over time, however, the definitions and the respective measures of youth PA self-efficacy have become more and more heterogeneous. Therefore, Voskuil and Robbins [24] conducted a concept analysis regarding the defining attributes, antecedents and consequences of the different conceptualisations. Eventually, they defined youth PA self-efficacy “as a youth’s belief in his/her capability to participate in PA and to choose PA despite existing barriers” [24]. The conceptualization of self-efficacy regarding PA by Dishman and colleagues [25, 26] considers the two main points of this definition by addressing both the self-perceived confidence in the capability to be physically active as well as the recognition of barriers to PA [24].

To date, no instruments exist which are specifically constructed and appropriately validated to examine PA self-efficacy of early adolescents in secondary school in Germany. Questionnaires specifically designed for early adolescents are needed, especially regarding the wording of items. Twelve-year-olds produce a response quality worse than that of youths aged fourteen [27]. Scott [28] even argues that adolescents cannot answer properly to adult items before the age of sixteen. Furthermore, thorough validations of instruments assessing PA self-efficacy are generally scarce [29] and specific PA-related risk groups are even more rarely used in the validation of these scales [10, 12, 30].

Therefore, the purpose of this study was to validate a German version of the physical activity self-efficacy scale [26] using a sample of female sixth-graders. Because of the clustered nature of the data (students in classes), the validation was conducted in accordance with the multi-level approach described by Huang [31]. When dealing with individuals nested in groups, the use of multi-level modelling is strongly recommended [32, 33] as the assumption that individual perceptions are independent of one another cannot be maintained [34]. A violation of this assumption can lead to biased parameter estimates, false inferences regarding the psychometric properties and finally wrong conclusions about the reliability and validity of a scale [35, 36]. Therefore, factor structure and scale dimensionality were analysed by means of a multi-level confirmatory factor analysis (MCFA). Internal consistency was also estimated for both the individual and group level, respectively. Furthermore, criterion validity was tested by examining the relation of PA self-efficacy and actual PA on both levels.

## Methods

### Participants

The sample included 507 female sixth-graders recruited from 33 single-gender physical education (PE) classes of fifteen secondary schools in Munich. The participants were part of the CReActivity project, a randomized controlled trial aiming to promote PA of female sixth graders [37]. Mean age was 11.61 years (SD = .55, N = 430). The girls were on average of normal weight (mean BMI = 19.49, SD = 3.68, N = 386). The number of BMI values was diminished as parts of the sample refused to be weighed. Refusal was shown by both apparently overweight and normal weight girls. The sample comprised participants from households of low, medium and high socioeconomic status (SES; mean = 49.80, SD = 15.96, N = 412). SES was assessed by asking the adolescents to name and describe their parents’ current jobs. The answers were classified referring to the International Socioeconomic Index of occupational status (ISEI), which is based on the International Standard Classification of Occupation 2008 (ISCO-08) [38]. When the jobs of both parents could be classified, the job with the higher ISEI was considered (HISEI). Vague answers making a definite classification impossible, reduced the number of HISEI values.

The study was approved by the ethics commission of the Technical University of Munich (155/16 S) and the Ministry of culture and education of the state of Bavaria in Germany.

### Measures

#### Self-efficacy

The physical activity self-efficacy scale was used to assess the girls’ perceived self-efficacy to be physically active [26]. The scale contains eight items. The original items were validated in samples of sixth- and eighth-grade girls. Confirmatory factor analyses supported a unidimensional model [25, 26, 39]. Participants responded on a five point Likert-type scale ranging from 1 (“Disagree a lot”) to 5 (“Agree a lot”). The scale validated here was translated into German by means of a combined translation technique including the committee approach and the pretest procedure [40, 41]. The committee comprised four bilingual experts that translated the original scale into German. The main advantage of the committee approach lies in the possibility of correcting each other quickly and directly in the case of a mistake. Since it was necessary to not only translate the items but to adapt them in order to prevent the participants from misunderstanding the meaning of the items and thus guarantee content equivalence between the original and translated scale, the committee approach was deemed more useful than the classic back-translation technique. The pretest procedure implies a pilot study, which allows the identification of potential problems before start of the main study. A sample of 161 sixth graders (N_female_ = 71, N_male_ = 90) attending the same type of school was used for pilot testing to eventually be able to provide a final version that every student can understand.

#### Physical activity

To assess leisure time MVPA, participants wore accelerometers (ActiGraph GT3X - wGT3X-BT) for seven consecutive days except during water-based activities. The device was placed on the right hip. Sampling rate was set to 30 Hz. Participants had to wear the device on weekdays starting at the latest on their way to school until 9 pm or until they went to bed. On weekend days, the students had to put it on as soon as they woke up until 9 pm or until they went to bed.

### Procedures

Several weeks before the beginning of the data assessments, students and their parents were informed in writing about the purpose and the procedure of the assessment. Students did not participate unless they had provided a written consent form before.

Data assessments took place at the beginning of a physical education lesson. Codes were used to ensure the anonymity of the participants. Before handing out the accelerometers, the assessment team explained how to put them on. At least 25% of the students of each class received an information sheet on how to handle the accelerometers enabling them to serve as contact persons for their classmates. After the students had put on the accelerometers correctly, they filled out the questionnaire. The actual PE lesson did not start until the last student had completed the questionnaire.

### Data analysis

#### Multi-level validation of the physical activity self-efficacy scale

As the sample examined in this study provides clustered data, the validation is based on the multi-level approach by Huang [31]. Ignoring the clustered nature of the data can lead to wrong parameter estimates, standard errors and model fits. It is recommended to account for multi-level data even if intracluster correlations (ICC) of the single manifest variables are small (e.g., ICC = 0.01) [35, 42]. In nested data, factor structures might not be the same for each level [31]. MCFA provides the opportunity to examine individual- and group-level data simultaneously. To this end, the total population covariance matrix is divided into a pooled within-group covariance matrix and a between-group covariance matrix. Thereby, both within- and between-group effects can be estimated at the same time. Huang [31] offers an R syntax to be used with the lavaan package [43] and a function for generating the required matrices based on the five MCFA steps outlined by Hox (44, Chapter 14).

In step 1, a single-level factor analysis is performed using only the pooled within-group covariance matrix. In step 2, the null model, which assumes the factor structure of step 1 for both levels, is fitted. In this step, both the pooled within- and between-group covariance matrices are used as input. Equality constraints for the two levels are applied, meaning that factor loadings, variances and covariances for every manifest variable and latent factor are assumed to be the same for the two levels. In step 3, new group-level latent variables are introduced to estimate the variance attributed to the groups. This step is referred to as the independence model since the newly introduced group-level variables are not allowed to covary. This constraint is eliminated in step 4, the so-called saturated model. All degrees of freedom at the between-group level are now used, making it a fully saturated model. Finally, in step 5, the model that is actually hypothesized, is specified. At least one overall general factor is added for the between-group level which is defined responsible for the correlation of the latent group-level factors [31]. For every model, small negative residual variances on the class level are fixed to zero to allow the model to fully converge. This common practice is particularly required when the number of units on the group level is small and ICCs are close to zero [44].

To evaluate model fit, several fit indices were considered [45]: the χ2-likelihood ratio statistic, the comparative fit index (CFI), the Tucker-Lewis index (TLI), the root mean square error of approximation (RMSEA) and the standardized root mean square residual (SRMR). As the χ2-goodness of fit test tends to reject reasonably fitting models when applied to data of large samples, a variety of fit indices was used to estimate model fit [45]. Whereas CFI and TLI values greater than .95 indicate a good model fit, values less than or equal to .08 suggest a good model fit when RMSEA and SRMR are considered [45].

Furthermore, as fit tends to improve by including more variables in the model, parsimony is another criterion taken into account when deciding for a preferred model. Akaike’s information criterion (AIC) was considered as it not only compares the fit of different models but also penalizes an increasing amount of estimated parameters [46]. The AIC is a relative fit index which is used for model comparison. Lower AIC values indicate better model fit. Eventually, the aim is to generate a model that explains as much variance as possible with as few variables as necessary. Therefore, the optimal combination of model fit and parsimony is sought [47].

Scale reliability is indicated by Cronbach’s alpha. Values were calculated for both levels separately by using the alpha function of the psych package in R [48]. In case of non-positive definite matrices, alpha was calculated for the nearest positive definite matrix [49].

Criterion validity was examined by correlating self-efficacy values with the participants’ MVPA values. Pearson r is indicated for both the pooled within-group correlation and the between-group correlation.

Model-based correlations were used to estimate potential relations between latent factors.

#### Physical activity

During the download of the PA data, the vector magnitude counts were summed over 1-s epochs (10-s epochs for GT3X because of lower memory and battery capacities). The low frequency extension filter was not used. Wear-time validation was conducted with the algorithm by Choi, Liu, Matthews and Buchowski [50]. A participant’s PA data was considered valid if data of at least three weekdays and one weekend day were available with at least eight hours of wear time being required for a valid day. The wear-time validated PA data was analysed utilizing the cut points by Hänggi, Phillips and Rowlands [13] to eventually calculate the average duration of MVPA per day for each participant. The cut points by Hänggi et al. [13] were chosen because they provide a precise assessment and were validated by applying the same data sampling and processing criteria as the ones chosen for this study [51].

## Results

Of the 507 participants originally included in the sample, 53 had missing values in at least one item of the physical activity self-efficacy scale. The values were missing completely at random. Additionally, the substantial sample size, the moderate interitem correlations and the acceptable proportion of missing values allowed for an available item analysis (AIA). Given these circumstances, an AIA leads to equivalent results compared with a multiple imputation analysis, which makes it unnecessary to intervene and replace missing values [52]. The participants excluded from the analysis did not differ significantly from the valid sample regarding BMI, SES, self-efficacy and MVPA. Finally, 454 sixth-graders built the final sample.

The descriptive statistics of the eight items of the physical activity self-efficacy scale are presented in Table 1. Means of the items ranged from 3.17 (*SD* = 1.30) to 3.96 (*SD* = 1.14). Skewness and kurtosis values were low to moderate. ICCs were small (< .05).

**Table 1:** Descriptive statistics for the items of the physical activity self-efficacy scale (N = 454)

Item #	M	SD	Skewness	Kurtosis	ICC
1	3.83	1.06	−0.49	− 0.65	.02
2	3.36	1.35	−0.30	−1.10	.04
3	3.96	1.14	−0.92	0.03	.04
4	3.86	1.10	−0.66	− 0.30	.03
5	3.33	1.30	−0.19	−1.06	.03
6	3.64	1.21	−0.53	−0.65	−.01
7	3.71	1.10	−0.42	−0.69	−.01
8	3.17	1.30	0.00	−1.11	.01

*Note. M* mean; *SD* standard deviation; *ICC* intraclass correlation

For the single-level one-factor model, an acceptable fit was found (model A in Table 2). Compared to model A, the null model (χ2 = 85.87, CFI = .975, TLI = .975, RMSEA = .048, SRMR = .047, AIC = 10,425.47) fit better regarding the TLI and RMSEA, but fit worse when considering the CFI, SRMR and AIC. Whereas model fit did not change substantially for the independence model (χ2 = 75.50, CFI = .977, TLI = .973, RMSEA = .050, SRMR = .044, AIC = 10,431.10), the fit of the saturated model differed according to the respective fit index (χ2 = 44.36, CFI = .979, TLI = .942, RMSEA = .073, SRMR = .030, AIC = 10,455.96). In the last step of the algorithm outlined by Hox (44, Chapter 14) and Huang [31], model B was obtained, see Table 2. For this model, one overall general factor was added for the class level (1 × 1-model). Model B contains twice as many degrees of freedom as the single-level model A, which led to an increase of the χ2 and AIC value. However, according to the CFI, TLI and RMSEA, model fit improved compared to the single-level model A. For model B, all factor loadings were significant on the individual level whereas on the class level three out of eight items exhibited significant loadings (Table 3).

**Table 2:** Fit indices for five models tested via Multilevel Confirmatory Factor Analysis

	Model				
Fit index	A: One factor, single-level, 8 items	B: One factor, multi-level, 8 items	C: Two factors, multi-level, 8 items	D: One factor, single-level, 6 items	E: One factor, multi-level, 6 items
χ2	44.36	61.36	56.94	26.58	32.10
df	20	40	38	9	18
CFI	.977	.982	.984	.980	.986
TLI	.968	.975	.976	.967	.976
RMSEA	.054	.049	.047	.068	.059
SRMR	.032	.038	.035	.030	.035
AIC	9624.68	10,432.96	10,432.54	6957.92	7537.17

*Note. df* degrees of freedom; *CFI* comparative fit index; *TLI* Tucker-Lewis index; *RMSEA* root mean square error of approximation; *SRMR* standardized root mean square residual; *AIC* Akaike’s information criterion

**Table 3:** Standardized factor loadings and correlations of latent factors

Model						
	A	B	C		D	E
Items	F1	F1	F1	F2	F1	F1
Individual level						
1	**.72**	**.73**	**.72**		**.72**	**.73**
2	**.45**	**.45**		**.51**	x	x
3	**.64**	**.65**	**.64**		**.65**	**.65**
4	**.66**	**.68**	**.67**		**.66**	**.66**
5	**.49**	**.49**		**.55**	x	x
6	**.61**	**.60**	**.60**		**.60**	**.60**
7	**.76**	**.76**	**.76**		**.78**	**.77**
8	**.76**	**.76**	**.76**		**.76**	**.75**
Class level						
1		**.65**	**.91**			.48
2		**.82**		**.84**		x
3		.12	.57			.58
4		.33	.49			1.03
5		**1.04**		**.96**		x
6		.30	.27			.09
7		.32	.36			**.52**
8		.17	.43			.55
R_individual_ (F1, F2)			**.87**			
R_class_ (F1, F2)			.69			

*Note*. Values in bold are statistically significant (*p* < .05); x = item not included in the model

For model C (2 × 2-model), a second latent factor was introduced on both levels which is modelled by items 2 (in the original version by Dishman and colleagues [26]: “I can ask my parent or other adult to do physically active things with me.”) and 5 (original item: “I can ask my best friend to be physically active with me during my free time on most days.”). Responses to these two items rather depend on the social environment of the early adolescents and not solely on themselves. The idea of creating a separate factor comprising these two items was further supported as they exhibited the lowest factor loadings in the single-level model A (Table 3). In line with this, their correlations with the other items were below average. Specifying two factors on each level for model C decreased the number of degrees of freedom because two additional parameters had to be estimated compared to model B. However, model C had a better model fit with respect to each index, including the AIC (Table 2). Furthermore, model C also showed a better model fit compared to its single-level counterpart (χ2 = 41.50, CFI = .979, TLI = .969, RMSEA = .053, SRMR = .030, AIC = 9623.81). In model C, six out of eight items exhibited factor loadings close to or above .50 on the class level, two items had loadings lower than .40. The model-based correlation of the latent factors in model C was 0.87 on the individual level and 0.69 on the class level (Table 3).

In a final step, the items 2 and 5 were excluded to test for the unidimensional structure of a six-item scale both in a single- and multi-level analysis. Again, the multi-level model (model E) fit the data better than its single-level counterpart (model D). Furthermore, model E exhibited the best fit of all models with respect to the CFI and TLI indices (Table 2). Like in every other model, the items of model E showed significant factor loadings on the individual level. On the class level, five out of six items had loadings close to or above .50, yet only one loading was statistically significant (Table 3).

Cronbach’s alpha for the eight-item scale was 0.84 on the individual level and 0.91 on the class level. In the two-factor solution, the six-item subscale exhibited an alpha value of 0.85 on the individual level and 0.90 on the class level. Cronbach’s alpha values for the items 2 and 5 were 0.44 on the individual level and 0.96 on the class level, using the nearest positive definite matrix for the class level.

Average MVPA per day was 80.44 min (SD = 21.01, N = 374). The pooled within-group correlation between average MVPA per day and self-efficacy measured by the eight-item scale was 0.19 (p < .001, N = 345). Correlations with the six-item subscale (r = 0.19, p < .001, N = 350) and two-item subscale (r = 0.14, p < .01, N = 359) were similar. Considering the 33 classes on the group level, the between-group correlations of MVPA per day and self-efficacy measured by the eight-item scale was r = 0.65 (p < .001, N = 33). Correlations of MVPA with the first (r = 0.57, p < .001) and the second subscale (r = 0.59, p < .001) were comparable.

## Discussion

The guidelines for PA [5] are only fulfilled by a minority of children, adolescents and adults (e.g., 12, 15). As individual PA behaviour is often sustained from adolescence to adulthood (e.g., 9), interventions trying to enhance PA of children and adolescents are of great importance. To improve young people’s PA behaviour, individual self-efficacy is one of the most important determinants to focus on (e.g., 20, 21). The physical activity self-efficacy scale [26] assesses the individual self-efficacy regarding PA of adolescents and incorporates the findings of the concept analysis of Voskuil and Robbins [24].

In this study, a German version of the physical activity self-efficacy scale was validated in terms of its factorial validity, internal consistency and criterion validity. Self-efficacy does not only differ on the individual level but also on the group level. Therefore, and because the scale was validated with clustered data, analysis was conducted based on a multi-level framework [31]. This way, a mismatch between the constitution of self-efficacy and its assessment and analysis was circumvented. The physical activity self-efficacy scale can be applied to measure the construct both on the individual and the group level at the same time by applying the summary index model [53]. It suggests that the aggregated variable on the group level can be the sum or the average of a variable assessed at the individual level.

The examination of its factorial validity in this sample indicated that the physical activity self-efficacy scale not only measured PA self-efficacy with six items but also PA-related social support of family and friends with the two remaining items. The actual self-efficacy items build a highly reliable measurement. These findings applied both to the individual and class level. Furthermore, the scale provided substantial criterion validity as it contributed to the explanation of the female sixth-graders’ PA, especially on the class level.

Self-efficacy in our sample was comparable to the self-efficacy of the sample of sixth-graders used to validate the original scale by Dishman and colleagues [26] in terms of the means (3.61 vs. 3.74, see Table 1). Standard deviation was almost identical (0.83 vs. 0.79), kurtosis of the items was similar (− 1.10 to 0.03 vs. -1.05 to 0.65).

The fit of the single-level one-factor model A (Table 2) was acceptable, which justified the implementation of the subsequent steps of the MCFA. The ICCs of the items did not suggest a substantial variance between the classes. The fits of the null model, independence model, and saturated model did not allow for a clear-cut inference about a statistically significant group-level variance [31, 44].

Concerning the fit indices which are less sensitive to the number of parameters to be estimated, the fit of the one-factor multi-level model B was better than the fit of the single-level model A (see Table 2). This result justifies a MCFA as it shows that there was relevant between-group variance, which should be taken into account, although the ICCs of the items were low.

The introduction of a second factor on both levels (model C) further improved model fit. Whereas six items of the physical self-efficacy scale by Dishman and colleagues [26] indeed relate to PA-related self-efficacy, the wording of the items 2 and 5 addresses the family and peers of the participant as agents providing social support for PA. This interpretation can be traced back to the original self-efficacy scale by Saunders and colleagues [54], which built the foundation for the scale validated in this study. This scale comprised the three subscales barriers, positive alternatives and support seeking, which item 2 and 5 were part of. The answers to these items mainly depend on circumstances which cannot be fully controlled by an early adolescent. If the parents both work full time and, on top of that, are not interested in being physically active, the child lacks the means to change these circumstances. Similarly, if the best friend does not like to be active and cannot be reached within a manageable distance for a child, chances of regularly engaging in PA together are low. Thus, an actually self-efficacious adolescent can disagree with these items while agreeing with the remaining items, which refer to more personally controllable aspects and attitudes. The fact that items 2 and 5 show the lowest loadings in the single-level model A and exhibit comparatively low correlations with the remaining items indicate that this scenario occurred in a considerable number of cases. Thus, in the sample used in this study, the items selected by Dishman et al. [26] do not form a unidimensional scale. Taken together, these findings argue against the supposed unidimensional structure of the physical activity self-efficacy scale [25, 26, 39].

The sample of this study only included adolescents attending schools in the city of Munich. Living in an urban area with good infrastructure, they have good opportunities to visit their friends on their own by foot, bike or public transport. In a sample including students both from urban and rural areas, the possibilities of visiting the best friend on one’s own might differ largely between classes. In this case, between-group variance specifically concerning item 5 would increase. The fact that model C fits the data better than its single-level two-factor counterpart means that even with this rather homogeneous sample, there is variance regarding both factors on the individual as well as on the class level. This again underlines the benefit of the multi-level approach used in this study. Using only a single-level approach would have led to a loss of substantial information regarding PA self-efficacy and PA social support on the class level.

Bandura [55] posited four main sources of self-efficacy. Verbal persuasion by influential others saying that one has the capabilities to master the task ahead can increase self-efficacy. The current emotional and physiological state also plays a role, as an energetic and healthy person will most likely perceive a higher self-efficacy compared to a self-conscious person dealing with a serious health condition. The two most important resources, however, are mastery and vicarious experiences. The experience of mastering a particular challenge should increase one’s confidence to also master similar tasks in the future. Vicarious experiences could finally explain the finding that the two latent factors PA self-efficacy and PA social support are highly correlated (r ≥ 0.69, Table 3). It can be assumed that people who regularly provide social support for PA are physically active themselves, which is implied in the wording of items 2 and 5. Thus, they can serve as role models for a healthy PA behaviour. The concept of vicarious experience [55] suggests that if a person observes another person performing successfully, it can enhance the confidence in the own ability to succeed in the same task, especially when the person being observed is deemed similar to oneself. This can lead to the effect that an adolescent’s PA behaviour influences his/her best friend and vice versa. Hence, vicarious experience [55] might mediate the association of PA social support and PA self-efficacy. Furthermore, the attraction paradigm [56] proclaims that perceived similarity to a peer is a major factor that determines whether a relationship turns into a close friendship or not. Taken together, it can be assumed that close friends often think the same way about being physically active because their similarity led to their friendship in the first place [56] and vicarious experiences help to further assimilate to each other in terms of PA self-efficacy [55]. This could explain the correlation of item 5 with the six items assessing PA self-efficacy.

Since the perceived similarity between observer and role model plays a major role in vicarious experiences [55] and adolescents normally perceive their parents as being less similar to them as their friends, it is unlikely that vicarious experiences explain the association of parental PA support and children’s PA self-efficacy. Instead, parental PA support might have a direct positive effect on PA self-efficacy [57]. In sixth-graders, particularly parents’ emotional and instrumental social support have an effect on the adolescents’ PA self-efficacy [58]. These findings could explain why responses to item 2 correlate highly with self-efficacy.

Given these points, although previous validations of the physical activity self-efficacy scale supported a unidimensional model [25, 26, 39], the present study shows the need to distinguish a second factor assessing PA-related support by parents and peers with regard to statistical and conceptual aspects. Additionally, it is worth mentioning that the previous single-level validation studies revealed factor loadings below 0.40 for at least one item [25, 39]. As it has been criticized elsewhere [30], this indicates a lack of scale homogeneity and questions a unidimensional structure, however, these results have not been discussed appropriately [25, 39].

Finally, if the actual goal is to measure early adolescents’ PA self-efficacy, items 2 and 5 should be excluded from the data assessment, as other researchers have done [59]. Consequently, specifying a one-factor structure on both levels after excluding the items 2 and 5 led to the best overall model fit (model E), especially with respect to the CFI and TLI indices. Furthermore, the comparison between the single-level and multi-level analysis of this shortened version of the physical activity self-efficacy scale [26] also supported the consideration of between-group differences.

Reliability was estimated for the individual and the class level separately [31]. Cronbach’s alpha for the eight-item scale was good on the individual level and excellent on the class level [60]. Cronbach’s alpha is positively associated with the number of items [61]. Alpha values for the shorter six-item subscale representing PA self-efficacy, however, were not diminished, which speaks for an even higher internal consistency of this sub-group of items compared to the complete scale. Cronbach’s alpha for the two-item support factor was low on the individual level and excellent on the class level. Thus, the association of support from family and peers becomes less ambiguous when the nesting of students in classes is considered. Composite reliability was also estimated to make sure that reliability was not underestimated when using Cronbach’s alpha [61, 62]. Differences between the two methods were marginal. Higher reliability values on the group level were expected since reliability tends to increase and measurement error tends to decrease when measures are aggregated across students within the same classes [63].

Likewise, the use of aggregated measures on the class level normally affects factor loadings and correlations to be higher on this level [63]. This assumption was only partially met (Table 3). Although the number of classes included in this study fulfils the minimum amount for conducting a MCFA [64], it still might have reduced the group-level factor loadings and model-based correlations between the latent factors.

Finally, the scores of the complete scale and its subscales were correlated with actual PA to evaluate the criterion validity. The average MVPA level was in line with a systematic review including 36 studies mainly conducted in Europe and North America [65]. However, average MVPA was higher than in previous German studies. It is unlikely that the sample in this study exhibits an unrepresentatively good PA behaviour. In fact, the differences to previous German studies can be explained by the use of different PA measurement instruments (self-report questionnaires vs. accelerometers) and different sampling and analysis decisions concerning the accelerometer data, which have a severe impact on the estimated PA values [10, 14, 51, 66]. In this study, a high resolution was chosen, leading to the most accurate PA estimates possible [13, 50, 51], which at the same time implicated higher MVPA values than usually found in Germany. The participants’ scores on the complete eight-item scale and the two subscales revealed a significant positive relation to their actual PA. This is in line with previous research emphasising the role of self-efficacy as an important determinant of healthy PA behaviour of children and adolescents (e.g., 19, 20). The correlations were clearly higher on the class level, which again justifies the multi-level approach and underlines the differentiation between self-efficacy on the individual and on the group level. Furthermore, this could favour the incremental value of multi-level modelling regarding the association between self-efficacy and PA. Considering that the construct of PA self-efficacy is by definition closely connected to the actual PA behaviour [24], the correlation between PA self-efficacy and actual PA is rather low in the majority of studies (e.g., 20, 21, 22). The higher reliability and lower measurement error of the aggregated class-level measures used here, could contribute to detecting correlation coefficients that are closer to the respective true value [63].

### Strengths and limitations

The main strength of this study lies in the application of a multi-level approach to clustered data of students nested in classes. Even though the ICCs suggested a negligible variance on the class level [35, 42], multi-level models consistently exhibited a better fit and thus are more suited to depict the actual data. By means of the multi-level approach, it was shown that reliability and criterion validity of the validated scale can differ significantly between the individual and the class level.

The findings should be verified in a more diverse sample comprising girls and boys of different age and from both rural and urban background. Further research on the construct or, more specifically, the physical activity self-efficacy scale [26] should include a larger number of classes on the group level and also more students per class. Measurement invariance across time should be tested in a longitudinal design with a sample that is not exposed to any intervention. Additionally, validation of the scale in a sample with low PA would further support the applicability of the scale to adolescents with diverse activity levels.

## Conclusions

Thoroughly validated scales with good psychometric criteria are essential for sound evaluations of cross-sectional studies and intervention programmes. This multi-level validation suggests that the German version of the physical activity self-efficacy scale [26] not only measures PA self-efficacy but also PA-related social support by family and friends. The two latent factors are highly correlated on both levels, but statistically and conceptually distinguishable. Therefore, it should be discussed if the scale should continue to be considered unidimensional.

This study argues for the validation of psychometric scales using a multi-level approach because substantial information regarding class-level self-efficacy would have been lost by applying a single-level validation.

It is recommended to exclude the social support items from data assessments to have a highly reliable and valid measurement instrument for individual- and class-level PA self-efficacy.

## Supplementary information

**Additional file 1.** validation data; description: minimal dataset necessary to replicate the analysis.

## Data Availability

All data analysed during this study are included in the supplementary information files of this article.

## Abbreviations

AIC: Akaike’s information criterion
BMI: Body mass index
CFI: Comparative fit index
df: degrees of freedom
HISEI: Highest International Socioeconomic Index of occupational status
ICC: Intracluster correlation
ISCO: International Standard Classification of Occupation
ISEI: International Socioeconomic Index of occupational status
M: Mean
MCFA: Multi-level confirmatory factor analysis
MVPA: Moderate-to-vigorous physical activity
PA: Physical activity
PE: Physical education
RMSEA: Root mean square error of approximation
SD: Standard deviation
SES: Socioeconomic status
SRMR: Standardized root mean square residual
TLI: Tucker-Lewis index
WHO: World Health Organization
